# Clinical Utility of Delta Lactate for Predicting Early In-Hospital Mortality in Adult Patients: A Prospective, Multicentric, Cohort Study

**DOI:** 10.3390/diagnostics10110960

**Published:** 2020-11-17

**Authors:** Pablo del Brio-Ibañez, Raúl López-Izquierdo, Francisco Martín-Rodríguez, Alicia Mohedano-Moriano, Begoña Polonio-López, Clara Maestre-Miquel, Antonio Viñuela, Carlos Durantez-Fernández, Miguel Á. Castro Villamor, José L. Martín-Conty

**Affiliations:** 1 Advanced Life Support Unit, Emergency Medical Services, 40002 Segovia, Spain; pdelbrioibaanezd@saludcastillayleon.es; 2 Emergency Department, Hospital Universitario Rio Hortega, 47012 Valladolid, Spain; rlopeziz@saludcastillayleon.es; 3 Advanced Clinical Simulation Centre, Faculty of Medicine, Universidad de Valladolid, Advanced Life Support Unit, Emergency Medical Services, 47005 Valladolid, Spain; 4 Faculty of Health Sciences, Universidad de Castilla la Mancha, 45600 Talavera de la Reina, Spain; Alicia.Mohedano@uclm.es (A.M.-M.); Begona.polonio@uclm.es (B.P.-L.); Clara.maestre@uclm.es (C.M.-M.); Antonio.vinuela@uclm.es (A.V.); Carlos.durantez@uclm.es (C.D.-F.); JoseLuis.MartinConty@uclm.es (J.L.M.-C.); 5 Faculty of Medicine, Universidad de Valladolid, 47005 Valladolid, Spain; mcastrovi@saludcastillayleon.es

**Keywords:** prognosis, lactate clearance, biomarker, emergency medical services, emergency department, critical care

## Abstract

One of the challenges in the emergency department (ED) is the early identification of patients with a higher risk of clinical deterioration. The objective is to evaluate the prognostic capacity of ΔLA (correlation between prehospital lactate (pLA) and hospital lactate (hLA)) with respect to in-hospital two day mortality. We conducted a pragmatic, multicentric, prospective and blinded-endpoint study in adults who consecutively attended and were transported in advanced life support with high priority from the scene to the ED. The corresponding area under the receiver operating characteristics curve (AUROC) was obtained for each of the outcomes. In total, 1341 cases met the inclusion criteria. The median age was 71 years (interquartile range: 54–83 years), with 38.9% (521 cases) females. The total 2 day mortality included 106 patients (7.9%). The prognostic precision for the 2 day mortality of pLA and hLA was good, with an AUROC of 0.800 (95% CI: 0.74–0.85; *p* < 0.001) and 0.819 (95% CI: 0.76–0.86; *p* < 0.001), respectively. Of all patients, 31.5% (422 cases) had an ΔLA with a decrease of <10%, of which a total of 66 patients (15.6%) died. A lactate clearance ≥ 10% is associated with a lower risk of death in the ED, and this value could potentially be used as a guide to determine if a severely injured patient is improving in response to the established treatment.

## 1. Introduction

One of the challenges in the emergency department (ED) is the rapid identification of those patients who, upon arrival, may have a greater risk of clinical deterioration, which may lead to serious adverse events (SAE), such as unplanned admission to the intensive care unit (ICU), major adverse cardiovascular events, or early mortality [1].

Although there are a series of early warning scores based on different physiological parameters, which are capable of predicting the risk of deterioration in EDs [2,3], there are still situations in which SAEs could be detected earlier if there was an effective early warning [4].

Therefore, different biomarkers with prognostic value are being evaluated, such as lactate [5]. Under normal physiological conditions, lactate production remains constant with lactate consumption; prolonged hyperlactacidemia (serum concentrations > 4 mmol/L) is the result of an increase in production or a reduction in consumption [6]. Hyperlactacidemia is often caused by an imbalance between oxygen supply and demand, and therefore elevated lactate can be seen as a non-specific marker of tissue hypoxemia, with this being a documented risk factor for mortality in patients with a serious and, more specifically, an infectious pathology [7,8].

The predictive value of a single lactate measurement as an indicator of hypoxic cellular distress is being investigated [9], and even more so, to detect mortality beyond the first 24 h [10]. A second lactate measurement can help to quantify the change from the initial measurement, which is called delta lactate (ΔLA), with a direct relationship with mortality [11,12].

The measurement of lactate levels in the ED is a routine analytical procedure [13], and point-of-care testing is beginning to be implemented in emergency medical services (EMS) [14]. Therefore, at this time we have a high level of evidence of the prognostic value of lactate, both in the ED and in the prehospital setting [5,15,16].

The primary objective of this study was to evaluate the prognostic capacity of ΔLA (correlation between prehospital lactate (pLA) and hospital lactate (hLA)) with respect to early in-hospital mortality (up to two days from the index event). The secondary objective was to analyse the predictive capacity of ΔLA for 7 and 30 day in-hospital mortality.

## 2. Experimental Section

### 2.1. Study Design and Setting

We conducted a pragmatic, multicentric, prospective and blinded-endpoint study in adults who consecutively attended and were transported in advanced life support (ALS) with high priority from the scene to the ED between the 1 October 2018, and 30 November 2019.

The study was carried out by six ALSs who transferred patients to five hospitals of the public health system (Burgos University Hospital, Segovia Hospital Complex, Salamanca University Assistance Complex, Rio Hortega University Hospital and Valladolid University Clinic), with a reference population of 1,351,962 inhabitants.

EMS operates non-stop 24/7 every day. Requests for assistance are evaluated by a physician at the emergency coordination centre who determines the most appropriate resource based on care needs. The ALS is made up of a physician, an emergency registered nurse (ERN) and two emergency technicians. On the scene or en route, they perform standard advanced life support actions according to the protocols for each pathology. Patients are transferred by the ambulance team to the ED. In the triage area, an ERN determines the level of priority and then hospital care begins.

This study was approved by the Research Ethics Committee of all participating centres (reference REC: #PI 18-010, #PI 18-895, #PI 2018-10/119 and #CEIC 2049) dated 9 March 2018. The study protocol is available online (doi.org/10.1186/ISRCTN17676798); we follow the STROBE guidelines for reporting. All patients (or guardians) signed the informed consent, including consent to data sharing. The ERN of the ALS attempted to obtain informed consent. If the patient’s clinical situation or level of consciousness did not allow this, an ED physician tried again to obtain consent. In situations, such as death, or patients referred to the ICU in which it was not possible to obtain the document, a relative or legal guardian was contacted to ensure that informed consent was obtained.

### 2.2. Selection of Participants

A patient was considered to meet the criteria to be included in the study if they had been evaluated and transferred by an ALS to the ED of the referral hospital and did not meet any exclusion criteria, among which are: under 18 years of age, presence of cardiorespiratory arrest, death prior to or during transport, pregnant women, patients with an acute psychiatric pathology or those with a documented terminal illness. Those which were also excluded from the initial cohort were those who, even meeting the inclusion criteria, had not undergone a hospital lactate analysis or those who had not been able to complete follow-up, due to lack of data or duplication. If a patient was admitted more than once during the study period, only the first admission was counted. In cases in which informed consent was not obtained despite multiple attempts, the case was excluded.

### 2.3. Outcome Measures and Study Protocol

The main outcome variable was in-hospital mortality within 48 h from any cause, and secondary in-hospital mortality at 7 and 30 days was also analysed.

### 2.4. Study Protocol and Collection of the Parameters

A procedure was developed for the determination of pLA, the operation of the equipment, cleaning, maintenance and calibration and specific training was carried out for all members of the EMS. The traceability of all the test strips used in the study has been monitored, by checking the expiration, serial number and lot number.

For the data collection, a standardised form was designed (medical history routinely used by EMS), where the ALS physician recorded demographic variables (age and gender), standard vital signs and prospectively the pLA value. All the prehospital clinical data analysed refer to the team’s first contact with each of the patients. In the ambulance or on the scene, a venous blood sample was obtained with which pLA was determined. The analysis was performed using the Accutrend^®^ Plus meter (Roche Diagnostics, Mannheim, Germany). All the measuring devices were calibrated every 100 determinations, always by the same researcher from each ALS, using Accutrend^®^ BM-Control-Lactate control solution (Roche Diagnostics, Mannheim, Germany).

During the first hour of ED care, a new blood test was performed on those patients who required it, and hLA was determined together with the rest of the standard analytical parameters. Thirty days after the index event, an associate researcher from each hospital, by reviewing the electronic medical record (JIMENA-SACYL), the hospital outcomes were obtained: hLA value, need for admission and/or ICU, data from 2, 7 and 30 day in-hospital mortality, days of admission and diagnosis.

With the two lactate measurements, clearance was calculated according to the usual formula for the established time [17,18,19],

(1) $$\mathrm{Lactate}\;\mathrm{clearance}\;(\%)\;=\;\frac{initial\;lactate-Follow-up\;lactate}{Initial\;lactate}\times \;100$$

### 2.5. Statistical Analysis

The database was designed and organised after the collection of double-entry data in order to reduce transcription errors. To guarantee the correct traceability of patients between the prehospital setting and hospital care, the link criteria between the EMS history and the hospital electronic history were the date, ALS code, time of arrival at the ED, patient affiliation, gender and age. Prior to statistical analysis, the database was cleaned using logical tests and range tests (detection of extreme values). The presence and distribution of unknown (non-existent) values in all the variables evaluated were verified. The case registration form was tested to remove ambiguous elements and to protect the data collection instrument. The process was robust and consistent. Statistical analyses were performed using XLSTAT software (New York, NY, USA) for Microsoft Excel version 14.4.0 ((Microsoft Inc., Redmond, WA, USA), and SPSS 20.0 (SPSS Inc^®^, Chicago, IL, USA).

Continuous quantitative variables are described with the median and interquartile range (IQR). Qualitative variables are described with absolute and relative frequencies (%). To compare the group, in the quantitative variables whose distribution did not show evidence of differing from normal distribution, the Student’s *t*-test was used, otherwise the Mann–Whitney U test was used. To compare the percentages, the chi-square test was used for the 2 × 2 contingency tables or, in the case of a low frequency being observed, in some cells of the corresponding table, Fisher’s exact test.

Survival analyses were performed using the Kaplan–Meier method and the Cox proportional hazard function.

In-hospital mortality statistics refer to mortality rates at 48 h, patients who were discharged “alive” within 48 h were considered “alive” for the purposes of this analysis. The secondary outcomes were defined as death within 7 days and 30 days of hospital admission. From these estimates, the corresponding area under the receiver operating characteristics curve (AUROC) was obtained for each of the outcomes.

In all the tests carried out, a confidence level of 95% and a value of *p* < 0.05 were considered significant.

## 3. Results

### 3.1. Patient Baseline

**Figure 1:**
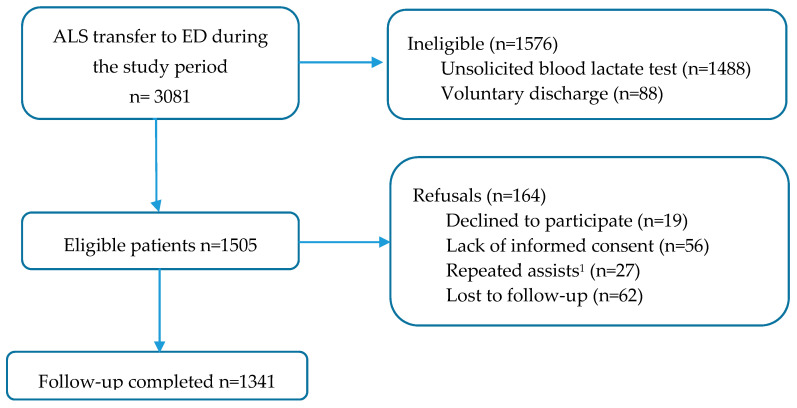
Flowchart of the participants in the study. ^1^ In the case of more than one attendance at the emergency department, only the first attendance was analysed. ALS: advanced life support; ED: emergency department.

In total, 1341 cases met the inclusion criteria (out of a total of 3081 patients assessed by EMS) and were part of the cohort analysed (see Figure 1).

The median age was 71 years (IQR: 54–83 years), with 38.9% (521 cases) females. The 2 day mortality was 106 patients (7.9%), while it rose to 158 patients (11.8%) at 7 days and 229 patients (17.0%) at 30 days. Regarding the pathologies that the patients included in the study present, it has been observed that the most prevalent diagnosis has been that of cardiovascular origin (29.3%, 393 cases) followed by neurological problems (17.4%, 234 cases), with the ICU admission rate from the ED at 21.3% (285 cases) (see Table 1).

There is a significant correlation between the pLA and hLA levels with two day in hospital mortality. For both values, the median for this mortality range was 5.5 mmol/L (IQR: 4.4–7.6 and 3.3–8.0 mmol/L), while the median in survivors was 3.3 mmol/L (IQR: 2.2–4.8 mmol/L) for pLA and 2.1 mmol/L (IQR: 1.4–3.5 mmol/L) for hLA.

### 3.2. Prognostic Accuracy of pLA and hLA

**Figure 2:**
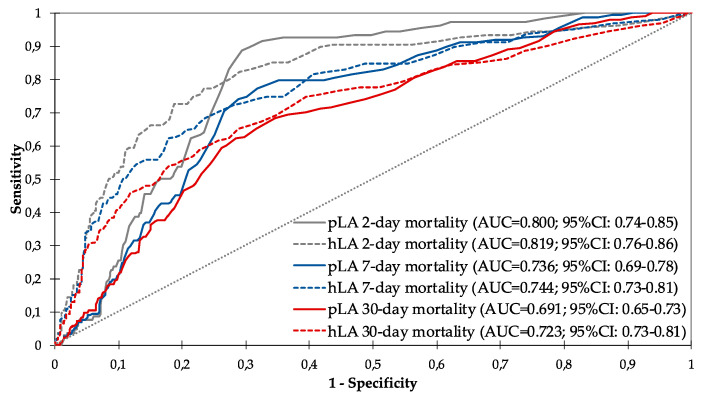
Diagnostic performance curves and areas under the curve with 95% confidence intervals for pLA and hLA for 2, 7 and 30 day mortality (in all cases *p* < 0.001). pLA: prehospital lactate; hLA: hospital lactate; AUC: area under the curve; CI: confidence interval.

The prognostic accuracy of the 2 day mortality of pLA and hLA was good, with an AUROC of 0.800 (95% CI: 0.74–0.85; *p* < 0.001) and 0.819 (95% CI: 0.76–0.86; *p* < 0.001), respectively. Both pLA and hLA lose predictive capacity as time passes. (see Figure 2).

### 3.3. ΔLA and Risk Stratification

The patients were classified into two groups taking as reference the results of lactate clearance, stratifying the ΔLA in a group with clearance < 10% and another with clearance ≥ 10%. Of all patients, 31.5% (422 cases) had ΔLA with a decrease of <10%, of which a total of 66 patients (15.6%) died. In contrast, in the group with ΔLA ≥ 10%, mortality was only 40 patients (4.4%) (*p* < 0.001) for 2 day mortality (Table 2).

Similarly, and also in line with previous studies, the raw lactate values were segregated based on the initial value and the cut-off point was established at 2 mmol/L to make two comparison groups and measure mortality. With a pLA value < 2 mmol/L, 253 patients (18.9%) were counted, with a single death among them. Altogether, 1088 patients (81.1%) had reference values ≥ 2 mmol/L and in this case the death toll rose to 105 (9.6%). Considering the hLA values with the same cut-off points, 596 (44.4%) and 10 deaths (1.7%) were found with <2 mmol/L. We counted 745 patients (55.6%), of which 96 died (12.9%), with ≥2 mmol/M (see Table 2).

**Figure 3:**
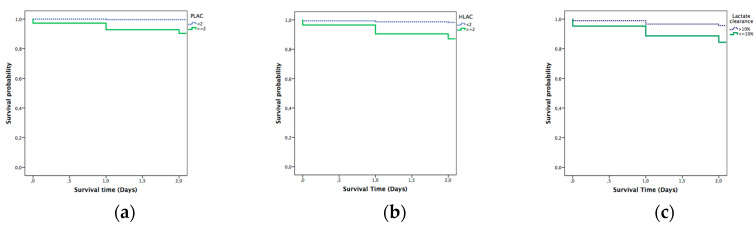
Kaplan–Meier analysis for 2 day mortality: (**a**) prehospital lactate; (**b**) hospital lactate; and (**c**) lactate clearance. PLAC: prehospital lactate; HLAC: hospital lactate.

**Figure 4:**
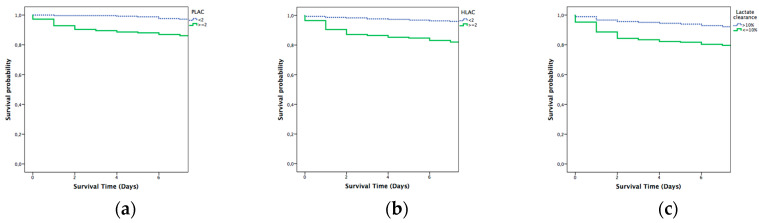
Kaplan–Meier analysis for 7 day mortality: (**a**) prehospital lactate; (**b**) hospital lactate; and (**c**) lactate clearance. PLAC: prehospital lactate; HLAC: hospital lactate.

**Figure 5:**
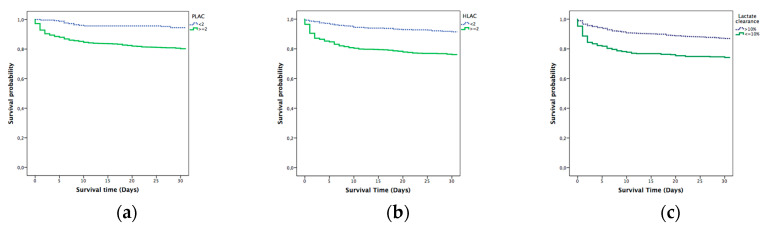
Kaplan–Meier analysis for 30 day mortality: (**a**) prehospital lactate; (**b**) hospital lactate; and (**c**) lactate clearance. PLAC: prehospital lactate; HLAC: hospital lactate.

The Kaplan–Meier analysis confirmed significantly longer in-hospital survival at 2 days in patients with lactate ≤ 2 mmol/L compared with patients with higher levels. Survival rates are also consistent with previous results after the analysis at 7 and 30 days. The differences between the survival curves were statistically significant (*p* = 0.001) (Figure 3, Figure 4 and Figure 5).

## 4. Discussion

With this study we observed that the measurement of ΔLA can be a quick and easy tool for determining the initial state and the short-term prognosis of a critical patient in an ED. Our results show that both a low lactate level (below 2 mmol/L) and a lactate clearance of more than 10% from the first prehospital determination to the second in the ED is related to an increase in survival.

The concept of lactate clearance was introduced at the end of the last century by Vincent et al. [20] and just as the temporal evolution of lactate and its elimination during resuscitation, this concept has been widely studied in different settings and clinical contexts [21,22].

To our knowledge, this is the first study that analyses the concept of early lactate clearance, in less than one hour, with data collected during prehospital care in the ambulance and in the ED [23,24]. The normalisation of lactate measured in relation to its clearance (ΔLA) was shown to be associated with a lower risk of early death in ED. Poor relative clearance of lactate is an excellent predictor of the risk of early mortality, ahead of the alteration of vital signs [25]. Thus, the measurement of lactate clearance can add useful information for the clinical management of critical patients in an ED.

In line with our results, different authors have observed how a decrease in the lactate level is associated with longer survival (24) and a good response to established treatment [26]. Specifically, Wada et al. and Bhat et al., verified that a decrease in lactate levels among patients attending the ED is associated with longer survival [27,28]; Gotmaker et al. studied the lactate clearance 6 h after the initial determination, obtaining data consistent with ours and asserting that the establishment of this practice can be a very effective tool for assessing the prognosis of critical patients [29]. Something similar is observed by Hguyen and Soliman who analysed this clearance over a longer period such as 12 or 24 h [30,31].

Our study does not only support these previous findings, but also assesses the behaviour of the cohort, with respect to a clearance cut-off point established at 10% of the initial lactate value. This same cut-off has been established by other authors where the elimination of ≥10% lactate at 6, 24 and 48 h is an independent factor related to mortality, even after adjusting for critical status. Lactate clearance is a direct influence factor on survival, more significant than the initial or maximum lactate level reached, in critically ill patients [32,33]. Ladha et al. studied patients admitted to the ICU with a lactate clearance ≥ 10% with respect to the initial value after 6 h, all of whom required less ventilatory support, less need for vasopressor therapy and had a shorter hospital stay [34]. More recent studies showed a higher probability of survival when a second lactate level concentration was less than 3.7 mmol/L, or with a relative lactate clearance ≥ 8% [35].

The first lactate determination in the prehospital setting (pLA) should be complemented with another in-hospital measurement (hLA) upon arrival of the patient. The assessment of ΔLA could help with decision making, reducing the subjectivity of the health worker and complementing the presence of abnormal vital signs [30]. The observation that there has not been a clearance of ≥10% of lactate or the presence of hyperlactacidemia above 2 mmol/L should make us think that perhaps greater intensity should be applied in terms of the resuscitation treatment that we provide. Infected patients with lactate between 2 and 4 mmol/L have a mortality risk that is twice that of patients with a lactate level less than 2 mmol/L [15]. The early identification of these patients at risk will allow us to improve our response with a reduction in the time of both the necessary diagnostic tests, as well as the establishment of effective treatment [36].

### Limitations

Our study has several limitations. Firstly, the study is subject to duration bias, as there was no specific protocol to guide the intervals at which lactate levels are drawn (i.e., time to baseline and time to repeat lactate level). This factor cannot be controlled and could have been delayed for various reasons inherent to the medical activity itself, which would confuse the results with an overestimation of the benefit of screening, although the one hour interval has always been respected. Secondly, the results could be affected by selection bias, since sicker patients with differences in clinical signs may lead to a different response to treatment. However, we did not find significant differences between the elimination group ≥ 10% and the elimination group < 10%, which means that the sample had a severity at the time of lactate extraction. Finally, for future studies, it would be advisable to record in what form and time the treatment is administered, and to explain the differences observed in mortality, perhaps the timing of these interventions is key in the ED.

## 5. Conclusions

In summary, lactate clearance in the initial moments of ED care appears to be a more reliable prognostic index than a baseline lactate value taken alone. Lactate clearance ≥10% is associated with a lower risk of death in the ED; this value could potentially be used as a guide to determine if a severely injured patient is improving in response to established treatment. Thus, the measurement of lactate clearance appears to be a quick and easy-to-implement tool to determine the initial status and prognosis of the critical patient in an ED. Having the ability to stratify a risk at the earliest stage in critically ill patients can help the ED to more effectively manage the care that these patients need to improve their outcomes.

## Figures and Tables

**Table 1:** Demographic, prehospital and hospital clinical outcomes.

Characteristic	Total	2 Day Mortality	*p* Value	7 Day Mortality	*p* Value	30 Day Mortality	*p* Value
Number (*n* (%))	1341 (100)	106 (7.9)		158 (11.8)		228 (17.0)	
Age (years)	71 (54–83)	78 (64–87)	<0.001	78 (66–87)	<0.001	78 (65–87)	<0.001
Female	521 (38.9)	41 (38.7)	0.970	62 (39.2)	0.915	88 (38.6)	0.931
pLA (mmol/L)	3.3 (2.2–4.8)	5.5 (4.4–7.6)	<0.001	4.9 (3.9–7.0)	<0.001	4.6 (3.1–6.9)	<0.001
hLA (mmol/L)	2.1 (1.4–3.5)	5.5 (3.3–8.0)	<0.001	4.7 (2.5–7.6)	<0.001	3.8 (2.3–6.8)	<0.001
Inpatients	899 (67.0)	106 (100)	<0.001	158 (100)	<0.001	228 (100)	<0.001
ICU admissions	285 (21.3)	60 (56.6)	<0.001	84 (53.2)	<0.001	113 (49.6)	<0.001
Pathology Group							
Circulatory	393 (29.3)	35 (33.0)		50 (31.6)		63 (27.6)	
Respiratory	144 (10.7)	7 (6.6)	0.189	11 (7.0)	0.409	25 (11.0)	0.338
Digestive	96 (7.2)	7 (6.6)	0.865	9 (5.7)	0.847	15 (6.6)	0.276
Neurology	234 (17.4)	11 (10.4)	0.448	24 (15.2)	0.866	38 (16.7)	0.442
Trauma	115 (8.6)	15 (14.2)	0.894	20 (12.7)	0.716	26 (11.4)	0.334
Poisoning	100 (7.5)	3 (2.8)	0.116	5 (3.2)	0.157	7 (3.1)	0.086
Infectious	212 (15.8)	26 (24.5)	0.697	35 (22.2)	0.413	49 (21.5)	0.456
Others	47 (3.5)	2 (1.9)	0.128	4 (2.5)	0.174	5 (2.2)	0.064

Values expressed as the total number (fraction) and medians (25th percentile–75th percentile) as appropriate. Patients included in previous mortality days were also considered for the next period of mortality. The *p* values were calculated with the Mann–Whitney U-test (age, pLA and hLA). The *p* values were calculated with the chi-square test (gender, inpatients, ICU admission and pathology). Other pathology: endocrine, genitourinary, diseases of the blood and the immune system. pLA: prehospital lactate; hLA: hospital lactate; ICU: intensive care unit.

**Table 2:** Correlation between ΔLA and 2 day mortality.

Characteristic	Total ^1^	Survivors ^1^	2 Day Mortality ^1^	*p* Value
Lactate clearance				
ΔLA < 10%	422 (31.5)	356 (84.4)	66 (15.6)	
ΔLA ≥ 10%	919 (68.5)	879 (95.6)	40 (4.4)	<0.001
Prehospital lactate				
<2 mmol/L	253 (18.9)	252 (99.6)	1 (0.4)	
≥2 mmol/L	1088 (81.1)	983 (99.4)	105 (9.6)	<0.001
Hospital lactate				
<2 mmol/L	596 (44.4)	586 (98.3)	10 (1.7)	
≥2 mmol/L	745 (55.6)	649 (87.2)	96 (12.9)	<0.001

^1^ Values expressed as the total number (fraction). ΔLA: delta lactate.
